# Oral Mucositis in Oncopediatric Patients: MTX and MMP‐1, MMP‐8, MMP‐13 Gene Polymorphisms

**DOI:** 10.1111/odi.70195

**Published:** 2026-04-12

**Authors:** Nicole Januário Ribeiro, Larissa Helena Tissi, Mateus Tissot Escobar, Ana Maria Gondim Valença, Ricardo Lehtonen Rodrigues Souza, Naila Francis Paulo de Oliveira, Maria Cristina Leme Godoy Santos

**Affiliations:** ^1^ Department of Cell Biology University Federal of Paraná Curitiba Paraná Brazil; ^2^ Postgraduate Program in Decision Models and Health Federal University of Paraíba João Pessoa Paraíba Brazil; ^3^ Department of Genetics Federal University of Paraná Curitiba Paraná Brazil; ^4^ Department of Molecular Biology Federal University of Paraíba João Pessoa Paraíba Brazil; ^5^ Department of Cell Biology Federal University of Paraná Curitiba Paraná Brazil

**Keywords:** leukaemia, lymphoma, metalloproteinases, methotrexate, mucositis, polymorphisms

## Abstract

**Background:**

This study investigates the association between single‐nucleotide polymorphisms (SNPs) in the MMP‐1, MMP‐8, and MMP‐13 genes and the risk of oral mucositis development in paediatric patients with leukaemia and lymphoma who are undergoing methotrexate (MTX) treatment. MTX is associated with inflammatory effects and oxidative stress, which activate MMPs, enzymes responsible for degrading the extracellular matrix. This study aimed to investigate the association between MMPs polymorphisms (rs1799750, rs3025058, and rs2252070) with OM in the oncopediatric patients treated with MTX.

**Methods:**

Genomic DNA from 100 patients was extracted from saliva and genotypes were obtained by PCR‐RFLP. Genotype data were measured using the Chi‐square test. Haplotype estimation, Hardy–Weinberg equilibrium, linkage disequilibrium, multiple logistic regression analyses was conducted using SNPStats and with R. MCA performed with the packages FactoMineR and factoextra.

**Results:**

The results show that the MMP‐1 g.‐1607 G>GG, MMP‐8 g.‐799 C>T, and MMP‐13 g.‐77 A>G polymorphisms are associated with the occurrence of oral mucositis. The MMP‐8 g.‐799 C>T polymorphism was also associated with greater disease severity.

**Conclusions:**

The study highlights the importance of these MMPs polymorphisms as potential markers for predicting susceptibility to oral mucositis, suggesting that these data could help tailor treatments to minimise the occurrence and severity of mucositis.

## 1 Introduction

Oral mucositis (OM) is a frequent, painful, and debilitating inflammatory complication in patients undergoing chemotherapy, especially in oncopediatric patients, compromising quality of life and potentially limiting the continuation of cancer treatment (Braguês et al. 2024).

Methotrexate (MTX), widely used in the treatment of childhood leukaemias and lymphomas, is one of the main agents associated with the development of OM due to its cytotoxic potential and its ability to induce oxidative stress and inflammation (Driehuis et al. 2020; Filetici et al. 2024; Heil 2019).

MTX influences the upregulation of matrix metalloproteinases (MMPs) synthesis and gene expression, contributing to the development of OM (Cardoso et al. 2021). MMPs are endopeptidase enzymes that degrade components of the extracellular matrix and regulate inflammation and remodelling of tissue (Atanasova et al. 2023; Cardoso et al. 2021; Luchian et al. 2022; Singh; Singh, 2020). In OM, it is postulated that MMPs participate in activating various signalling pathways, recruiting inflammatory cells, inducing apoptosis, and causing mucosal damage by disruption of cell–cell and extracellular matrix‐cell interactions (Sonis 2004).

The development of OM depends not only on the cancer treatment regimen, dosage and number of cycles, but also on patient characteristics, including genetic polymorphisms. In the context of OM in children with hematologic malignancies, the literature has been focused primarily on genes related to folic acid metabolism—such as the MTHFR gene (which encodes the enzyme methylenetetrahydro folate reductase)—transport protein genes (ATP‐binding cassette family and solute carrier family), and miRNAs (Guimarães et al. 2025). However, the literature remains scarce regarding the relationship between genetic polymorphisms in the MMPs genes and OM.

Among all MMPs, collagenases (MMP‐1, 8, and 13) appear to play an important role in oral pathogenesis. MMP‐1 degrades different substrates, such as collagen I, II, III, aggrecan, entactin, gelatin, TNF precursor, among others present in the extracellular matrix (Flores et al. 2017; Lee et al. 2022; Nikolov et al. 2020), and its overexpression is associated with aggressive cancer cell growth (Kowalczyk et al. 2023). Additionally, the literature demonstrated that MTX increased the expression of MMP‐1 and reduced collagen production in strains of fibroblasts (Nabai et al. 2015). The MMP‐1 g.‐1607 G>GG (rs1799750) polymorphism alters transcriptional activity and can potentially increase the level of protein expression (Nishioka et al. 2000). This polymorphism has been correlated with different oral pathologies, such as periodontitis (Saremi et al. 2023), oral cancer (Li et al. 2018), and periapical dental lesions (Torres et al. 2020).

Different studies have pointed out the relationship between MMP‐8 and the triggering of inflammation in processes associated with the oral mucosa, due to its abundance in gingival connective tissues and salivary fluid (Baidya et al. 2024), and it may be an important biomarker for susceptibility to oral diseases (Batool et al. 2023; Xanthopoulou et al. 2023; Zhang et al. 2021). The MMP‐8 g.‐799 C>T (rs11225395) polymorphism alters protein expression and has been associated with peri‐implantitis (Fragkioudakis et al. 2025; Jin et al. 2025), oral submucous fibrosis (Qamar et al. 2025), periapical dental lesion (Evrosimovska et al. 2015), and periodontitis (Chou et al. 2011).

MMP‐13 degrades diverse types II, III, IV, and X collagens, being one of the most important enzymes in extracellular matrix remodelling (Leeman et al. 2002). The MMP‐13 g.‐77 A>G polymorphism (rs2252070) has been associated with oral pathologies such as chronic periodontitis (Prasanna et al. 2018), oral and oropharyngeal squamous cell carcinomas (de Matos et al. 2019), and dental caries (Çağırır Dindaroğlu et al. 2021).

It is essential to note that these polymorphisms have already been analysed in haplotypes (groups of alleles that are inherited together) and are associated with inflammatory conditions, like Dupuytren contracture (Rodrigues et al. 2024), posterior tibial tendinopathy (Baroneza et al. 2014), and oral implant loss (de Araujo Munhoz et al. 2019).

Thus, research increasingly highlights the involvement of MMPs in various oral diseases, suggesting that their use as biomarkers may be promising. Furthermore, the literature has frequently highlighted the relationship between MMPs and their inhibitors as possible mediators of OM, since these enzymes act in multiple pathways known to be positively stimulated in OM, leading to tissue damage and inflammation (Atanasova et al. 2023; Dai et al. 2024).

Investigations of polymorphisms in oncopediatric patients treated with MTX in the Brazilian population have already been conducted analysing 15 different polymorphisms—MTHFR rs1801133; ABCC2 rs717620; ABCG2 rs2231137 and rs2231142; SOD2 rs4880; CAT rs7943316; TNF‐α rs1800629; IL6 rs1800795; VDR rs1544410, rs2228570 and rs731236; DNMT1 rs2228611; DNMT3A rs7590760; DNMT3B rs6087990 and rs2424913—with 3 of these polymorphisms associated with OM (ABCG2 rs2231142, CAT rs7943316, VDR rs1544410) (Coêlho et al. 2022; de Souza et al. 2023; Viana Filho et al. 2021, 2023).

The role of MMPs is fundamental in the pathogenesis of chemotherapy‐induced OM. While the role of gelatinases (MMP‐2 and 9) is to maintain the integrity of the basal lamina, their dysregulation leads to the breakdown of this physical barrier and is closely linked to the release of pro‐inflammatory cytokines, which exacerbates the pathogenic mechanism of OM. Complementing this mechanism, collagenases are equally critical; MMP‐1, MMP‐8, and MMP‐13 are directly involved in the destruction of the extracellular matrix components that form the bulk of the oral mucosa connective tissue during the symptomatic phase of OM. Given their decisive role in irreversible structural damage, our study specifically focused on MMP‐1 g.‐1607 G>GG (rs1799750), MMP‐8 g.‐1612 5A > 6A (rs3025058), and MMP‐13 g.‐77 A>G (rs2252070), which are established markers, individually and in haplotype, for various inflammatory and destructive diseases.

Based on these facts, the present study aimed to investigate the association between MMP‐1 g.‐1607 G>GG (rs1799750), MMP‐8 g.‐1612 5A > 6A (rs3025058), and MMP‐13 g.‐77 A>G (rs2252070) polymorphisms, individually and in haplotypic combination, with OM in the same population of leukaemia and lymphoma oncopediatrics patients treated with MTX.

## 2 Materials and Methods

### 2.1 Study Population

This study was approved by the Ethical Committee in Research (n^o^. 4.878.034) and was carried out in accordance with the Declaration of Helsinki.

Paediatric patients diagnosed with leukaemia or lymphoma, treated with MTX and without oral inflammatory conditions prior to treatment were selected and divided into two groups: G1 (*n* = 16), which included individuals who did not present OM during chemotherapy treatment, and G2 (*n* = 84), composed of patients who developed OM during chemotherapy treatment, including 20 patients with mild or moderate mucositis (G2a) and 26 patients with severe mucositis (G2b). The remaining patients did not have their OM level determined in the study. Oral changes were evaluated by a previously calibrated team (kappa = 0.87) using the modified Oral Assessment Guide (OAG) (Cheng et al. 2004).

All participants were recruited from the northeast region of Brazil (Napoleon Laureano Hospital, João Pessoa, PB), and although the study sample was mostly composed of white people, as the Brazilian population is heterogeneous, it may have overlapping genotypes due to miscegenation.

Haematological data (haemoglobin, leukocyte, and platelet counts), biochemical data (urea nitrogen and creatinine), and cancer type were obtained from medical records and were collected at the time the patient developed OM. If the patient developed severe OM during follow‐up, data from that time point were selected. Haematological data collected from patients who did not develop OM corresponded to the last chemotherapy session.

Patients with no record of professional follow‐up by the oral care team during treatment, those in isolation or who were intubated or severely debilitated, and those treated with radiotherapy or both radiotherapy and chemotherapy were not included.

### 2.2 Genotyping

Oral mucosa cells were obtained by rinsing with 6 mL of sterilised 3% dextrose. Sample processing, as well as DNA extraction, was carried out as previously described (Aidar and Line 2007). The polymorphisms had previously been identified and included in the database of the National Centre for Biotechnology Information (http://www.ncbi.nlm.nih.gov/SNP/) with minor allele frequencies greater than 0.15. The MMPs genotypes were determined using the PCR‐RFLP assays. The primer sequences, PCR conditions and restriction enzymes are detailed in Table 1.

**TABLE 1:** Demographic and clinical data.

Data	No mucositis (G1 = 16)	Mucositis (G2 = 84)	Mild/moderate mucositis (G2a = 20)	Severe mucositis (G2b = 26)
Sex % (*n*)				
Female	69% (11)	36.9% (31)	40% (08)	46% (12)
Male	31% (05)	63.1% (53)	60% (12)	54% (14)
Average age	10.1 (±3.6)	10.4 (±4.9)	8.9 (±4.9)	10.8 (±4.6)
Cancer % (*n*)				
All	69% (11)	75% (63)	85% (17)	73% (19)
Others	31% (5)	25% (21)	15% (3)	27% (7)

*Note:* Others are acute myeloid leukaemia; acute promyelocytic leukaemia; chronic myeloid leukaemia; Hodgkin’s lymphoma; non‐Hodgkin’s lymphoma. Values are expressed in percentage, with the number of participants (*n*) in parentheses.

Abbreviation: ALL, acute lymphoblastic leukaemia.

PCR were carried out in a total volume of 15 μL containing approximately 100 ng of genomic DNA, 200 nmol of specific primers, and 1 unit of Go Taq Green PCR Master Mix (Promega Corporation, St. Madison, USA). A 10 μL aliquot of PCR products was then digested with 1 unit of specific enzyme (Restriction Enzymes Thermo Scientific—Fermentas Life Science—St. Lithuania, UE) overnight and was electrophoresed on a 5% agarose gel or 10% polyacrylamide gel and stained by GelRed Nucleic Acid Stain (Biotium Inc.—St. Fremont, USA) or silver nitrate, and the genotypes were identified according to the band patterns.

### 2.3 Statistical Analysis

Data were categorised and organised in a database to allow for analysis. Data normality was determined by the Kolmogorov–Smirnov test. Demographic and Clinical Data were measured using the chi‐squared test, Fisher's exact test, or the Mann–Whitney *U*‐test, adopting *α* < 0.05 in Jamovi 2.3.12 software (Stats Open Now, Sydney, Australia).

Genotype data of occurrence and severity measured using the Chi‐square test were applied to compare allele and genotype frequencies between groups. Statistical analysis of the haplotype estimation, linkage disequilibrium and Hardy–Weinberg equilibrium was conducted using SNPStats software (http://bioinfo.iconcologia.net/snpstats/start.htm) and confirmed using PHASE software (http://stephenslab.uchicago.edu/phase/download.html). The linkage disequilibrium and the multiple logistic regression analyses using codominant, dominant and recessive models and multiple correspondence analysis (MCA) were conducted with R (R Core Team 2025). MCA was performed with the packages FactoMineR (Le Sebastien et al. 2008) and factoextra (Kassambara and Mundt 2020).

## 3 Results

### 3.1 Demographic and Clinical Data

Males predominated in the sample population (57.8%). Furthermore, gender was significantly associated with the outcome of OM. Specifically, male patients had a 3.7‐fold increased chance of developing OM (OR = 3.71; 95% CI: 1.18–11.7; *p* = 0.019; chi‐square test).

The mean age was 10.3 years (±4.7). The primary diagnosis of acute lymphoblastic leukaemia predominated (74%), followed by other types (acute myeloid leukaemia 15%; non‐Hodgkin's lymphoma 6%; acute promyelocytic leukaemia 2%; chronic myeloid leukaemia 1%; Hodgkin's lymphoma 1%). No associations or differences were observed between the variables of age and underlying disease in relation to the occurrence and/or severity of OM. Table 1 shows the sex, average age and type of cancer of the participants in the groups.

There was a difference in leukocyte count (G1 = 5300 mm^3^ (3775–7725); G2 = 2700 mm^3^ (1100–5225); *p* = 0.006) and platelet count (G1 = 200,000 mm^3^ (147250–342,250); G2 = 120,000 mm^3^ (60250–241,150); *p* = 0.016) between the groups, by Mann–Whitney *U* test, with lower values being observed in individuals with OM. No associations with other haematological and biochemical parameters were detected (data not shown).

### 3.2 Genotype Data

The genotypic distributions were in Hardy–Weinberg equilibrium, except for MMP‐8 g.‐799 C>T (rs11225395) polymorphism, due to the large presence of the T/T allele in the mucositis group (G2) (Table 2).

**TABLE 2:** Hardy–Weinberg equilibrium in MMP‐1 g.‐1607 G>GG (rs1799750), MMP‐8 g.‐799 C>T (rs11225395), and MMP‐13 g.‐77 A>G (rs2252070) polymorphisms in groups.

Polymorphism	Group	*2G/2G*	*1G/2G*	*1G/1G*	*1G*	*2G*	*p*
*MMP‐1* rs1799750	All subjects	32	39	29	103	97	*p* = 0.029
	G2	29	35	20	93	75	*p* = 0.18
	G1	3	4	9	10	22	*p* = 0.11
*MMP‐8* rs11225395		C/C	C/T	T/T	C	T	
	All subjects	49	17	34	115	85	*p* < 0.0001
	G2	38	13	33	89	79	*p* < 0.0001
	G1	11	4	1	26	6	*p* = 0.430
*MMP‐13* rs2252070		*A/A*	*A/G*	*G/G*	*A*	*G*	
	All subjects	55	37	8	147	53	*p* = 0.61
	G2	51	27	6	129	39	*p* = 0.39
	G1	4	10	2	18	14	*p* = 0.61

*Note:* Values are expressed in the number of participants.

The present study confirmed that MMP‐1 g.‐1607 G>GG (rs1799750), MMP‐8 g.‐799 C>T (rs11225395), and MMP‐13 g.‐77 A>G (rs2252070) polymorphisms were related to the occurrence of OM in 100 participants with childhood leukaemias and lymphomas (Table 3).

**TABLE 3:** Polymorphisms frequencies in MMP‐1 g.‐1607 G>GG (rs1799750), MMP‐8 g.‐799 C>T (rs11225395), and MMP‐13 g.‐77 A>G (rs2252070) in oncopediatric patients without OM (G1) and with OM (G2).

Polymorphism	Without mucositis (G1)	Mucositis (G2)	*p*	OR (95% CI)
MMP‐1 g.‐1607 G>GG (rs1799750)				
Allele	*n* = 32	*n* = 168		
1G	68.75% (22)	41.1% (75)	*p* = 0.021	2.72 (1.21–6.11)
2G	31.25% (10)	58.9% (93)		
Genotype	*n* = 16	*n* = 84		
1G/1G	56.25% (09)	23.82% (20)	*p* = 0.032	4.11 (1.36–12.46)
1G/2G	25.00% (04)	41.66% (35)		
2G/2G	18.75% (03)	34.52% (29)		
MMP‐8 g.‐799 C>T (rs11225395)				
Allele	*n* = 32	*n* = 168		
C	81.25% (26)	53% (89)	*p* = 0.005	3.84 (1.50–9.82)
T	18.75% (06)	47% (79)		
Genotype	*n* = 16	*n* = 84		
C/C	68.75% (11)	45.2% (38)	*p* = 0.037	2.66 (1.85–8.33)
C/T	25.00% (04)	15.5% (13)		
T/T	06.25% (01)	39.2% (33)		
MMP‐13 g.‐77 A>G (rs2252070)				
Allele	*n* = 32	*n* = 168		
A	56.25% (18)	76.8% (129)	*p* = 0.028	2.57 (1.17–5.64)
G	43.75% (14)	23.2% (39)		
Genotype	*n* = 16	*n* = 84		
A/A	25.0% (04)	60.70% (51)	*p* = 0.031	4.63 (1.38–15.60)
A/G	62.5% (10)	32.15% (27)		
G/G	12.5% (02)	7.15% (06)		

*Note:* Values are expressed in percentage, with the number of participants (*n*) in parentheses.

Concerning the MMP‐1 g.‐1607 1G > 2G (rs1799750) polymorphism, there were significant differences in allele and genotype frequencies between groups. The allele 2G was found in 31.25% of the G1 and 58.9% of the G2 (*p* = 0.021; OR 2.72 (95% CI, 1.21–6.11)). The 2G/2G genotype was observed at a frequency of 18.75% in G1 and 34.52% in G2 (*p* = 0.032; OR 4.11 (95% CI, 1.36–12.46)).

As for the MMP‐8 g.‐799 C>T (rs11225395) polymorphism, the C allele was the most frequent in both groups, but a significant difference was found: 81.25% in the G1 and 53% in the G2 (*p* = 0.005; OR 95% 3.84 (1.50–9.82)). The T/T genotype showed a large difference between G1 (6.25%) and G2 (39.2%) (*p* = 0.037; OR 95% 2.66 (1.85–8.33)).

Considering MMP‐13 g.‐77 A>G (rs2252070) polymorphism, the A allele had a higher frequency in the G2 (76.8%) than in the G1 (56.25%) (*p* = 0.028, OR 95% 2.57 (1.17–5.64)). The most frequent genotype was A/G in the G1 (62.5%), whereas the A/A genotype was most frequent in the G2 (60.7%) (*p* = 0.031; OR 95% 4.63 (1.38–15.60)).

The relationship between polymorphisms and disease severity included 46 participants. Patients who were diagnosed with OM but lacked a specific grade of severity were excluded from this analysis. The results showed an association only between the allele of the MMP‐8 g.‐799 C>T (rs11225395) polymorphism and MO severity. The T allele had a higher frequency in the G2b (severe MO 67.3%) than in the G2a (mild/moderate MO 45%), suggesting that the T allele worsens the severity of the disease (*p* = 0.03; OR 95% 2.78 (1.18–6.54)) (Table 4).

**TABLE 4:** Polymorphisms frequencies in MMP‐1 g.‐1607 G>GG (rs1799750), MMP‐8 g.‐799 C>T (rs11225395), and MMP‐13 g.‐77 A>G (rs2252070) in oncopediatric patients with mild or moderate OM (G2a) and severe OM (G2b).

Polymorphism	Mild/moderate OM (G2a)	Severe OM (G2b)	*p* OR (95% CI)
MMP‐1 g.‐1607 G>GG (rs1799750)			
Allele	*n* = 40	*n* = 52	
1G	55% (22)	32.7% (17)	*p* = 0.053
2G	45% (18)	67.3% (35)	
Genotype	*n* = 20	*n* = 26	
1G/1G	25% (05)	15.4% (04)	*p* = 0.470
1G/2G	60% (12)	34.6% (09)	
2G/2G	3% (03)	50% (13)	
MMP‐8 g.‐799 C>T (rs11225395)			
Allele	*n* = 40	*n* = 52	
C	57.5% (23)	32.7% (17)	*p* = 0.030
T	42.5% (17)	67.3% (35)	2.78 (1.18–6.54)
Genotype	*n* = 20	*n* = 26	
C/C	50% (10)	27% (07)	*p* = 0.133
C/T	15% (03)	11.5% (03)	
T/T	35% (07)	61.5% (16)	
MMP‐13 g.‐77 A>G (rs2252070)			
Allele	*n* = 40	*n* = 52	
A	62.5% (25)	76.9% (40)	*p* = 0.202
G	37.5% (15)	23.1% (12)	
Genotype	*n* = 20	*n* = 26	
A/A	35% (07)	61.5% (16)	*p* = 0.136
A/G	55% (11)	30.8% (08)	
G/G	10% (02)	7.7% (02)	

*Note:* Values are expressed in percentage, with the number of participants (*n*) in parentheses.

**FIGURE 1:**
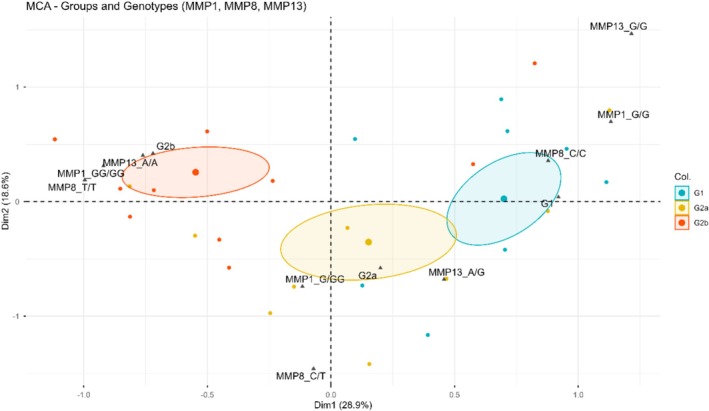
Multiple Correspondence Analysis (MCA) biplot showing the distribution of individuals according to group and genotypes (*MMP1*, *MMP8*, and *MMP13*). Individuals are coloured by group, and 95% confidence ellipses indicate the group dispersion. The proximity between categories and individuals reflects similarity in genotype profiles.

Although the genotypes did not show significant differences between groups G2a and G2b, in the MCA analysis we can see a separation of the 3 groups (Figure 1). The figure also shows that the heterozygous genotypes of the three genes are related to group G2a.

Polymorphisms used for haplotype inference were sorted according to their disposition on chromosome 11q22.3: MMP‐8, MMP‐1, and MMP‐13, respectively. Both groups had 5 haplotypes in common, and G2 had 3 exclusive haplotypes. The most frequent haplotypes were C‐1G‐A in G1 and T‐2G‐A in G2. Haplotype distribution analysis indicated no significant difference between the groups (Table 5).

**TABLE 5:** Haplotype frequencies considering polymorphisms MMP‐8 g.‐799 C>T (rs11225395), MMP‐1 g. 1607 and 1G > 2G (rs1799750), and MMP‐13 g.‐77 A>G (rs2252070), in oncopediatric patients without MO (G1) and with OM (G2).

Haplotypes	Total	G1	G2
T‐2G‐A	0.328	0.093	0.363
C‐1G‐A	0.206	0.375	0.172
C‐1G‐G	0.190	0.218	0.182
C‐2G‐A	0.137	—	0.168
T‐1G‐A	0.064	0.094	0.065
C‐2G‐G	0.042	0.219	0.007
T‐1G‐G	0.024	—	0.028
T‐2G‐G	0.008	—	0.014

*Note:* As in the analysis by genotypes, the MCA by haplotypes also shows a separation of the 3 groups (Figure 2).

## 4 Discussion

It is known that oral mucositis is a multifactorial disease and is associated with diverse factors, such as age, sex, haematological data, and genetic factors.

In most childhood cancers, males are more susceptible than females, and one of the most notable sex differences is detected in lymphomas and leukaemias (Taalas, et al. 2025; Liu et al. 2019), including in the Brazilian population (INCA 2022). In this study, we observed a male predominance in participants and in the occurrence of OM (G2). Globally, this male predominance in most cancers emerges in infancy, suggesting that some prenatal endogenous factors contribute to the sex disparity in cancer risk (Dunford et al. 2017; Liu et al. 2019). However, the underlying causes of sex differences in childhood cancers still require future investigation.

The overall toxicity profile of MTX, particularly in its high‐dose formulation, is well known in paediatric oncology. Its lack of cellular specificity results in collateral damage to healthy tissues, especially those with high proliferative activity, such as the bone marrow, gastrointestinal mucosa, liver, and kidneys. This off‐target toxicity can compromise treatment continuity and overall clinical outcomes. Gastrointestinal toxicity encompasses nausea, vomiting, diarrhoea, and, of particular relevance to our study, oral mucositis. Mucositis is often one of the earliest and most visible signs of systemic MTX toxicity, reflecting the drug's impact on mucosal cell turnover. Hematologic suppression is another central feature of MTX toxicity. Due to its immunosuppressive properties (particularly myelosuppression leading to neutropenia), affected patients experience a substantially increased risk of bacterial, viral, or fungal infections.

In our study, the findings were aligned with the expected toxicity spectrum of MTX. The group with OM (G2) had the lowest leukocyte and platelet counts, as pointed out by our previous studies in the same population (Damascena et al. 2020; Viana Filho et al. 2021). We suggested that a low leukocyte and platelet count may impair tissue recovery and exacerbate the inflammatory response to chemotherapy (Curra et al. 2022; Repsold and Joubert 2021). Furthermore, the cytotoxic capacity of MTX, leading to the death of epithelial cells and the OM development (Curra et al. 2021), associated with the myelosuppression generated by leukaemia and lymphoma treatments, may cause a high degree of OM, as observed in our study (84% of participants developed OM). However, no additional associations were found between OM and other hematologic or biochemical parameters in our analysis.

In the present study, we identified an important association between OM in childhood leukaemias or lymphomas treated with MTX and 3 polymorphisms in collagenase genes—MMP‐1 g.‐1607 G>GG (rs1799750), MMP‐8 g.‐799 C>T (rs11225395), and MMP‐13 g.‐77 A>G (rs2252070). These polymorphisms alter the transcriptional activity of MMPs; the 2G and T alleles are known to increase the transcriptional activity of the MMP‐1 and MMP‐8 genes (Decock et al. 2007; Rutter et al. 1998), while the G allele decreases the transcriptional activity of the MMP‐13 gene (Yoon et al. 2002). These alleles potentially alter the protein expression of MMPs, intensifying the degradation of the extracellular matrix and deregulating inflammatory signalling pathways.

Furthermore, increased the expression of MMP‐1 with used MTX (Nabai et al. 2015), and elevated MMP‐8 levels in patients undergoing radiotherapy, as observed by Brandt et al. (2025), corroborate the idea that MMP‐1 and MMP‐8 play a key role in the inflammatory response in oncologic contexts, and their overexpression may contribute to the worsening of oral mucosal lesions.

In contrast, the rs2252070 polymorphism in MMP‐13 showed a protective effect against severe oral mucositis, among individuals with the G/G and A/G genotype. The G variant appears to reduce MMP‐13 expression, resulting in less extracellular matrix degradation and reduced inflammation. This finding aligns with previous studies that indicated a protective role for rs2252070 in various oral conditions, such as dental caries and oral carcinoma (De Matos et al. 2019; Derqaoui et al. 2022). Although MMP‐13 is involved in tissue remodelling, it seems to play a more moderate role in matrix degradation compared to MMP‐1 and MMP‐8. Reduced MMP‐13 activity may help preserve the integrity of the oral mucosa during MTX treatment, decreasing the severity of ulcerative lesions. This protective mechanism may explain the observed effect of the G variant, which negatively modulates MMP‐13 expression, thus reducing the risk of additional mucosal damage (Prasanna et al. 2018; Roy et al. 2022).

When we analysed the relationship between these polymorphisms and the severity of mucositis, we observed a significant association only with the allele of the MMP‐8 g.‐799 C>T (rs11225395) polymorphism. The T allele had a higher frequency in patients with severe OM.

**FIGURE 2:**
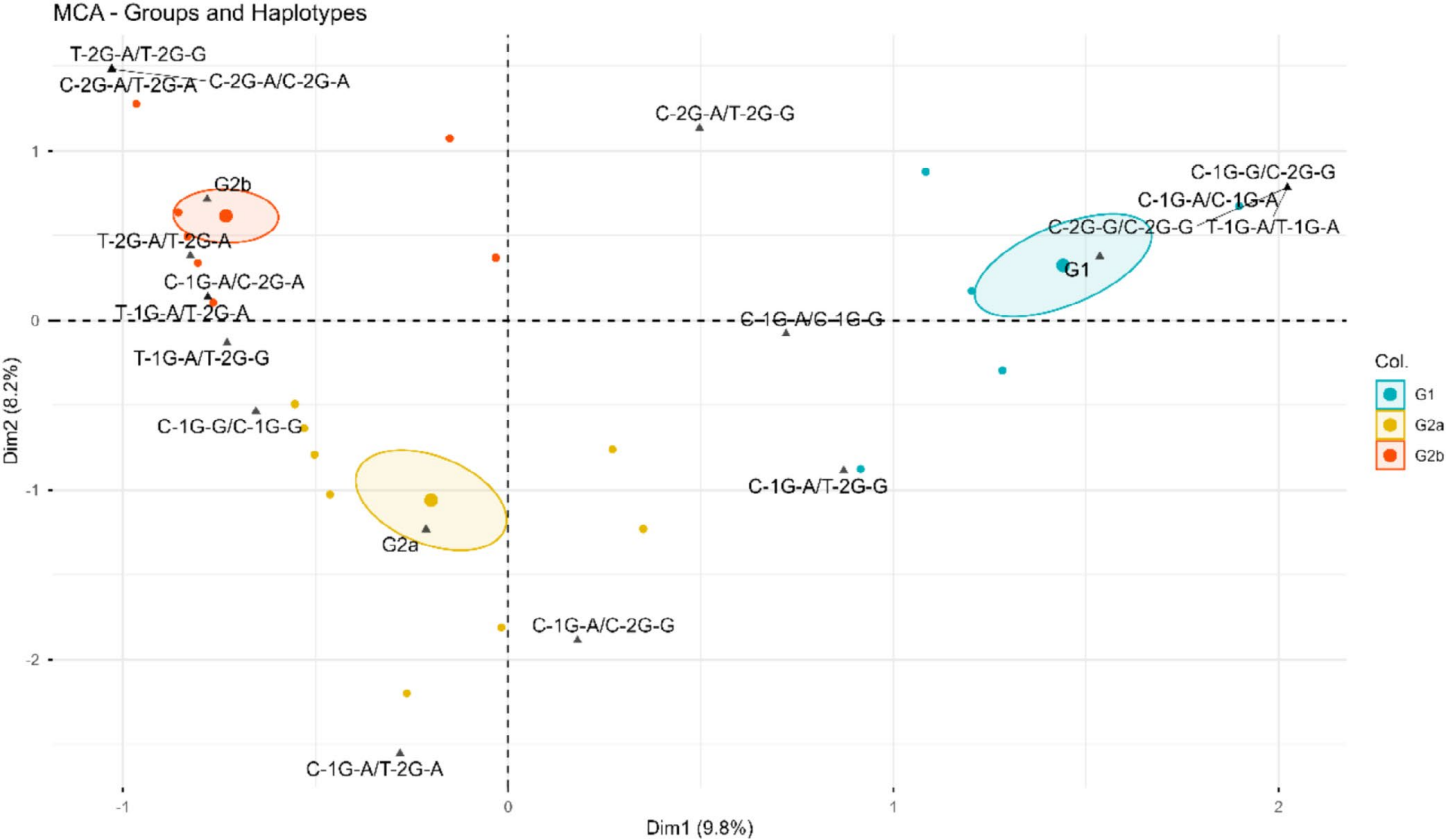
Multiple Correspondence Analysis (MCA) biplot showing the distribution of individuals according to group and haplotypes (*MMP8*, *MMP1*, and *MMP13*). Individuals are coloured by group, and 95% confidence ellipses indicate the group dispersion. The proximity between categories and individuals reflects similarity in haplotype profiles.

Haplotype distribution analysis indicated no significant difference between the groups; however, the MCA analysis separated the three groups. It is important to note that 11 different combinations were found among the participants. Few studies address this specific haplotype, but they also found a wide variety of haplotype combinations involving these polymorphisms. Although not statistically significant, we observed a trend toward a higher frequency of the C‐1G‐A haplotype in patients without MO, and the T‐2G‐A haplotype in patients with OM (Figure 2). It is also probable that the synergistic action of different polymorphisms increases the risk of OM in oncopediatric patients. Therefore, the analysis of haplotype frequencies seems to be of great value for understanding, treating, and preventing OM.

The current study provides new and unique results about the association of MMPs polymorphisms and MTX‐induced OM in childhood leukaemias and lymphomas. However, a critical analysis of the limitations is necessary. First, the study power is limited (< 80%) due to the small sample size, as this was a single‐center study based on an adverse event (OM) induced by a specific chemotherapy (MTX) in a childhood disease (haematological cancer). Furthermore, the study relied on medical records duly completed by hospital staff to ensure the inclusion of patients. For severity analysis, the lack of data regarding the level of mucositis limited the analysis to only 54.7% of the collected samples, thereby reducing statistical power. Another limitation is that the groups are not sex‐matched. This is a challenge inherent in clinical studies where patient recruitment is constrained by the disease prevalence within the specific population, leading to unavoidable imbalances in sex distribution. Even considering these limitations, the results together demonstrate an association between SNPs in the MMPs genes and the risk of OM development in paediatric patients with leukaemia and lymphoma who are undergoing MTX treatment. These results underscore the clinical relevance of genetic susceptibility in this population and suggest that further investigation into other MMP classes, particularly gelatinases (MMP‐2 and ‐9), is warranted to fully elucidate the proteolytic landscape of OM.

While many adverse drug reactions may be declining, mucosal damage remains an area of concern for chemotherapy. It is known that the functions of MMPs are difficult to predict; however, understanding the influence of MMPs and their polymorphisms on OM can bring significant benefits, not only as biomarkers and risk predictors, but also as a therapeutic alternative (Cabral‐Pacheco et al. 2020). For example, microcurrent therapy and low‐level laser therapy, used as supportive care in OM, have a notable impact on improving the wound healing process as they can significantly affect the expression levels of MMPs (El Makakey et al. 2025).

SNPs introduce a differential risk factor on top of the drug‐induced toxicity. Identifying patients with the high‐risk MMP genotypes enables clinicians to anticipate a more severe OM course and initiate prophylactic interventions earlier and more effectively, contributing to personalised medicine. The fact that even in a high‐dose MTX setting, genetic variations in the ECM remodelling pathway still influence the outcome demonstrates that the mechanism of tissue destruction is, in part, regulated by host genetics, offering targets for future pharmacological intervention. The polymorphism analysis identifies a subset of vulnerable individuals within the overall high‐risk population.

In conclusion, this work produces new insights into the molecular mediators that contributed to OM, suggesting that collagenases MMP‐1 g.‐1607 G>GG (rs1799750), MMP‐8 g.‐799 C>T (rs11225395), and MMP‐13 g.‐77 A>G (rs2252070) polymorphisms individually influence the occurrence of OM in leukaemias and lymphomas oncopediatrics patients treated with MTX.

## Author Contributions

**Nicole Januário Ribeiro:** investigation, methodology. **Larissa Helena Tissi:** methodology. **Mateus Tissot Escobar:** methodology. **Ana Maria Gondim Valença:** data curation, investigation. **Ricardo Lehtonen Rodrigues Souza:** writing – review and editing, formal analysis. **Naila Francis Paulo de Oliveira:** conceptualization, writing – review and editing, formal analysis. **Maria Cristina Leme Godoy Santos:** conceptualization, investigation, writing – original draft, formal analysis, supervision.

## Conflicts of Interest

The authors declare no conflicts of interest.

## Supporting information


**Table S1:** Population Data‐ Oncological children without history of oral mucositis.

## Data Availability

The data that support the findings of this study are available from the corresponding author upon reasonable request.
