# Supplementary material for: Oral Mucositis in Oncopediatric Patients: MTX and MMP‐1, MMP‐8, MMP‐13 Gene Polymorphisms
Source: Oral Dis. 2026 Apr 12;32(5):1440–50. doi: 10.1111/odi.70195 (PMC13364997; doi:10.1111/odi.70195)
Supplement: Supplementary file 1 — Table S1: Population Data‐ Oncological children without history of oral mucositis. [file ODI-32-1440-s001.docx]

**Table S1-** Population Data- Oncological children without history of oral mucositis.

| **Patient** | **Sex** | **Age** | **Patology** |
| --- | --- | --- | --- |
| G1-1 | F | 7 | Acute Lymphoblastic Leukaemia |
| G1-2 | F | 13 | Acute Lymphoblastic Leukaemia |
| G1-3 | M | 7 | Acute Myeloid Leukaemia |
| G1-4 | F | 15 | Acute Lymphoblastic Leukaemia |
| G1-5 | F | 7 | Acute Lymphoblastic Leukaemia |
| G1-6 | F | 10 | Acute Lymphoblastic Leukaemia |
| G1-7 | F | 17 | Acute Lymphoblastic Leukaemia |
| G1-8 | F | 13 | Acute Lymphoblastic Leukaemia |
| G1-9 | F | 14 | Acute Lymphoblastic Leukaemia |
| G1-10 | M | 8 | Acute Myeloid Leukaemia |
| G1-11 | M | 11 | Acute Myeloid Leukaemia |
| G1-12 | M | 12 | Acute Myeloid Leukaemia |
| G1-13 | F | 7 | Acute Lymphoblastic Leukaemia |
| G1-14 | F | 7 | Acute Lymphoblastic Leukaemia |
| G1-15 | F | 4 | Acute Lymphoblastic Leukaemia |
| G1-16 | M | 9 | Acute Myeloid Leukaemia |
| G2a-1 | M | 5 | Acute Lymphoblastic Leukaemia |
| G2a-2 | M | 17 | Acute Lymphoblastic Leukaemia |
| G2a-3 | M | 5 | Acute Lymphoblastic Leukaemia |
| G2a-4 | M | 9 | Acute Lymphoblastic Leukaemia |
| G2a-5 | F | 7 | Acute Lymphoblastic Leukaemia |
| G2a-6 | F | 8 | Acute Lymphoblastic Leukaemia |
| G2a-7 | M | 9 | Acute Myeloid Leukaemia |
| G2a-8 | F | 9 | Acute Lymphoblastic Leukaemia |
| G2a-9 | M | 19 | Acute Lymphoblastic Leukaemia |
| G2a-10 | M | 14 | Acute Lymphoblastic Leukaemia |
| G2a-11 | F | 15 | Acute Lymphoblastic Leukaemia |
| G2a-12 | M | 15 | Hodgkin's lymphoma |
| G2a-13 | M | 4 | Acute Lymphoblastic Leukaemia |
| G2a-14 | F | 10 | Acute Lymphoblastic Leukaemia |
| G2a-15 | M | 4 | Acute Lymphoblastic Leukaemia |
| G2a-16 | F | 3 | Acute Lymphoblastic Leukaemia |
| G2a-17 | M | 3 | Acute Lymphoblastic Leukaemia |
| G2a-18 | F | 5 | Acute Lymphoblastic Leukaemia |
| G2a-19 | F | 8 | Acute Lymphoblastic Leukaemia |
| G2a-20 | M | 8 | Acute Myeloid Leukaemia |
| G2b-1 | F | 17 | Acute Myeloid Leukaemia |
| G2b-2 | M | 3 | Acute Lymphoblastic Leukaemia |
| G2b-3 | M | 4 | Acute Lymphoblastic Leukaemia |
| G2b-4 | F | 15 | Acute Lymphoblastic Leukaemia |
| G2b-5 | F | 15 | Acute Lymphoblastic Leukaemia |
| G2b-6 | M | 17 | Acute Promyelocytic Leukaemia |
| G2b-7 | F | 16 | Acute Lymphoblastic Leukaemia |
| G2b-8 | F | 8 | Acute Lymphoblastic Leukaemia |
| G2b-9 | M | 7 | Acute Lymphoblastic Leukaemia |
| G2b-10 | M | 16 | Acute Lymphoblastic Leukaemia |
| G2b-11 | F | 10 | Acute Lymphoblastic Leukaemia |
| G2b-12 | M | 7 | non-Hodgkin lymphoma |
| G2b-13 | M | 12 | Acute Lymphoblastic Leukaemia |
| G2b-14 | F | 17 | Acute Lymphoblastic Leukaemia |
| G2b-15 | M | 7 | Acute Lymphoblastic Leukaemia |
| G2b-16 | F | 7 | Acute Promyelocytic Leukaemia |
| G2b-17 | M | 7 | Acute Lymphoblastic Leukaemia |
| G2b-18 | M | 10 | Acute Lymphoblastic Leukaemia |
| G2b-19 | M | 8 | Acute Myeloid Leukaemia |
| G2b-20 | F | 16 | Acute Lymphoblastic Leukaemia |
| G2b-21 | M | 14 | non-Hodgkin lymphoma |
| G2b-22 | M | 5 | non-Hodgkin lymphoma |
| G2b-23 | F | 10 | Acute Lymphoblastic Leukaemia |
| G2b-24 | M | 15 | Acute Lymphoblastic Leukaemia |
| G2b-25 | F | 5 | Acute Lymphoblastic Leukaemia |
| G2b-26 | F | 13 | Acute Lymphoblastic Leukaemia |
| G2-1 | F | 9 | Acute Myeloid Leukaemia |
| G2-2 | M | 4 | Acute Lymphoblastic Leukaemia |
| G2-3 | M | 8 | Acute Lymphoblastic Leukaemia |
| G2-4 | M | 18 | Acute Lymphoblastic Leukaemia |
| G2-5 | M | 6 | Acute Lymphoblastic Leukaemia |
| G2-6 | M | 6 | Acute Lymphoblastic Leukaemia |
| G2-7 | F | 19 | Acute Lymphoblastic Leukaemia |
| G2-8 | M | 10 | Acute Lymphoblastic Leukaemia |
| G2-9 | M | 19 | Acute Lymphoblastic Leukaemia |
| G2-10 | M | 8 | Acute Lymphoblastic Leukaemia |
| G2-11 | M | 10 | Acute Lymphoblastic Leukaemia |
| G2-12 | F | 6 | Acute Lymphoblastic Leukaemia |
| G2-13 | M | 5 | Acute Lymphoblastic Leukaemia |
| G2-14 | M | 19 | Acute Lymphoblastic Leukaemia |
| G2-15 | M | 19 | Acute Lymphoblastic Leukaemia |
| G2-16 | M | 10 | Acute Lymphoblastic Leukaemia |
| G2-17 | M | 19 | Acute Lymphoblastic Leukaemia |
| G2-18 | F | 12 | Acute Lymphoblastic Leukaemia |
| G2-19 | M | 11 | Chronic Myeloid Leukaemia |
| G2-20 | F | 15 | Acute Myeloid Leukaemia |
| G2-21 | F | 17 | Acute Lymphoblastic Leukaemia |
| G2-22 | F | 13 | non-Hodgkin lymphoma |
| G2-23 | M | 9 | Acute Myeloid Leukaemia |
| G2-24 | M | 7 | Acute Lymphoblastic Leukaemia |
| G2-25 | M | 5 | Acute Lymphoblastic Leukaemia |
| G2-26 | M | 7 | Acute Myeloid Leukaemia |
| G2-27 | M | 5 | Acute Lymphoblastic Leukaemia |
| G2-28 | M | 13 | Acute Lymphoblastic Leukaemia |
| G2-29 | M | 17 | non-Hodgkin lymphoma |
| G2-30 | M | 14 | non-Hodgkin lymphoma |
| G2-31 | M | 9 | Acute Myeloid Leukaemia |
| G2-32 | M | 15 | Acute Lymphoblastic Leukaemia |
| G2-33 | F | 15 | non-Hodgkin lymphoma |
| G2-34 | F | 7 | Acute Lymphoblastic Leukaemia |
| G2-35 | F | 6 | Acute Myeloid Leukaemia |
| G2-36 | M | 6 | Acute Lymphoblastic Leukaemia |
| G2-37 | F | 15 | Acute Lymphoblastic Leukaemia |
| G2-38 | M | 4 | Acute Lymphoblastic Leukaemia |

Patient: G1 = without mucositis group; G2a = mild/moderate mucositis group; G2b = severe mucositis group G2 = mucositis group (patients who OM severity undetermined); sex: F = female, M = male.
